# Machine learning to optimize additive manufacturing for visible photonics

**DOI:** 10.1515/nanoph-2022-0815

**Published:** 2023-03-17

**Authors:** Andrew Lininger, Akeshi Aththanayake, Jonathan Boyd, Omar Ali, Madhav Goel, Yangheng Jizhe, Michael Hinczewski, Giuseppe Strangi

**Affiliations:** Department of Physics, Case Western Reserve University, 2076 Adelbert Rd., Cleveland, OH 44106, USA; University of Calabria and CNR – Institute of Nanotechnology, Rende, CS, Italy

**Keywords:** additive manufacturing, machine learning, nanophotonics, physics-informed machine learning, two-photon polymerization

## Abstract

Additive manufacturing has become an important tool for fabricating advanced systems and devices for visible nanophotonics. However, the lack of simulation and optimization methods taking into account the essential physics of the optimization process leads to barriers for greater adoption. This issue can often result in sub-optimal optical responses in fabricated devices on both local and global scales. We propose that physics-informed design and optimization methods, and in particular physics-informed machine learning, are particularly well-suited to overcome these challenges by incorporating known physics, constraints, and fabrication knowledge directly into the design framework.

## 1 Introduction

The last few decades have seen major advances in nanophotonic technology to precisely control the wavefront of light. In this time, nanophotonic devices have taken on major roles in a wide range of fields including sensing [1, 2], communications [3], and energy [4], among others [5]. In large part, the growth of experimentally realised nanophotonic systems has been driven by the development and refinement of fabrication techniques [6]. To fully explore the design space of devices for manipulating light at the nanoscale, viable fabrication technologies must be capable of producing versatile and geometrically accurate 3D structures at dimensions well below the diffraction limit. Although there exists many fabrication techniques capable of fabricating 3D geometries at this scale, in practice the majority of nanophotonic fabrication is dominated by a few techniques [7].

**Figure 1:**
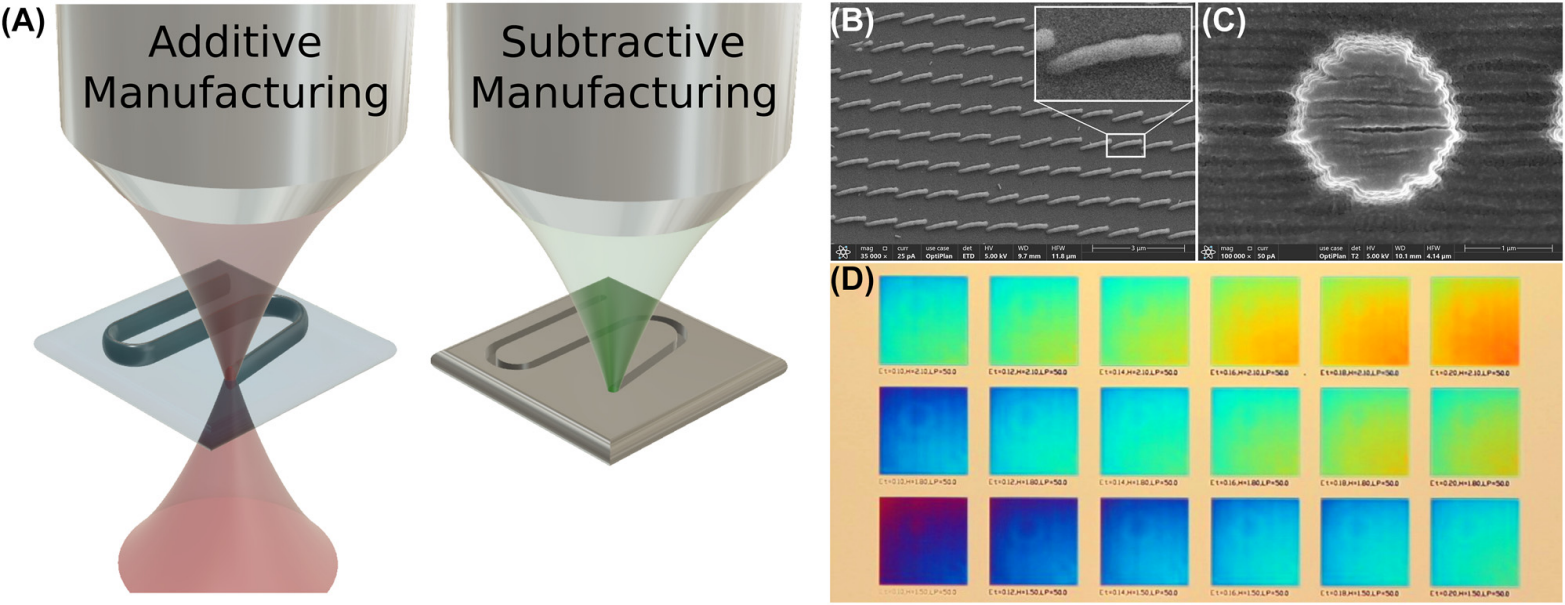
Comparison of additive and subtractive processes, and some examples of artifacts that can be introduced during the deposition process. (A) Schematic examples of manufacturing processes. Additive manufacturing (such as two-photon polymerization lithography (TPP)) deposits nanostructures in the final structural form. Subtractive manufacturing (such as focused ion beam lithography (FIB)) creates nanostructures by removing material from a larger bulk. (B) Scanning electron microscopy (SEM) image of an array of TPP deposited nano-pillars fabricated with multiple discrete layers. The side of the pillars is shown. The “bubbled” sidewall is caused by the voxel shape. (C) SEM image illustrating typical shrinking and striation effects observed during the TPP printing and deposition process. (B) and (C) Are examples of local TPP fabrication artifacts which affect each nanostructure individually. (D) Example of global fabrication artifacts in TPP. Each square is a regular array of Mie-resonant nano-pillars and should produce a constant color [16]. The diagonal color gradient in each square is indicative of a global change affecting multiple nanostructures throughout the array. Real color image in transmission, 10× objective.

The fabrication techniques relevant to visible nanophotonics can be broadly classified into subtractive manufacturing and additive manufacturing methods, illustrated in Figure 1A [8]. In subtractive manufacturing structures are created by directly removing material from a larger bulk or photoresist template [9]. Subtractive techniques include positive resist electron beam (e-beam) lithography or photolithography, focused ion beam (FIB) milling [10], and deep-UV lithography [11]. Subtractive techniques are common in 2D nanophotonics due to their relatively high resolution, which can regularly reach 10 nm [10]. However, there are several drawbacks to using subtractive methods for freeform 3D nanofabrication including design limitations due to beam path line-of-sight obstruction, and the typical necessity of multi-step fabrication processes [12]. Additionally, in subtractive manufacturing the structured medium is often the same material as the substrate. Utilizing a different material typically requires a more complex multi-step process.

Additive manufacturing techniques create nanoscale structures by directly depositing material in the final structural form, illustrated in Figure 1A. Some additive techniques include inkjet (electrohydrodynamic) printing, dip-pen nanolithography, and direct laser writing (DLW) [8, 13]. Negative tone e-beam lithography and photolithography can also be considered an additive technique. Additive methods have several desirable traits for nanofabrication including the capability to fabricate true freeform 3D structures typically avoiding beam obstruction during deposition, and reduction in the complexity of the fabrication to few or single-step processes. Since the material is directly deposited, structures can be fabricated with different material than the substrate without resorting to complex multi-step processes. Additionally, additive manufacturing allows for fast and flexible deposition and has the potential to reduce deposition waste – although in current practice nanolithography is typically wasteful with either method [14, 15].

Although additive manufacturing has many desirable aspects, limitations from current deposition technologies can restrict the application in nanophotonic fabrication. These complications can be local or global in scale and generally depend heavily on the particular process parameters [17]. Some examples of typical fabrication non-idealities encountered in TPP lithography (both local and global) are shown in Figure 1B–D. Local fabrication errors such as structural defects affect the optical resonance properties of single nanostructures, while global errors describe large-scale trends encompassing entire arrays of nanostructures. We propose that in many cases the effect of fabrication related limitations of additive manufacturing can be mitigated by utilizing appropriate simulation and design techniques which take into account the essential physics and geometrical constraints of the additive manufacturing processes. The recent growth of physics-informed optimization methods, in particular physics-informed machine learning, has opened new opportunities for exploring the complex relationship between photonic structure and optical response [18]. Physics-informed methods constructively utilize physical theoretical or experimentally observed information which is typically at best implicitly included and more often explicitly ignored in the optimization process. Incorporating this knowledge can lead to more highly optimized designs specifically attuned to additive manufacturing processes, with ensuing benefits in device performance.

## 2 Challenges in two-photon lithography

Two-photon polymerization lithography (TPP) is the most common additive manufacturing technique in visible nanophotonics, as well as one of the most developed, with over two decades of implementations [19–21]. TPP is a photochemical DLW process in which a femtosecond laser is focused into a small volume of photosensitive resin using a high numerical aperture objective [17, 22]. Full 3D fabrication is facilitated by moving the position of the laser focus with high resolution piezoelectrics. Deposition is accomplished by laser activation of a photoinitiator process which selectively polymerizes material in a small volume around the laser focus point. By harnessing the nonlinearity of the two-photon absorption, voxel dimensions of ∼100 nm can be routinely achieved [22, 23].

From a fabrication standpoint, TPP is desirable for cost, speed, scalability, and geometrical design flexibility within the requisite resolution for visible nanophotonics. Additionally, since the equipment requirements are typically less than for other lithography techniques, such as e-beam lithography, TPP has the potential for wider dissemination. However, barriers to widescale implementation still exist, predominantly resolution limits and the introduction of artifacts and errors through fabrication. Although ∼100 nm spatial resolution can be achieved via TPP much higher resolution (∼10 nm) is commonly required for many metasurface designs. This can be regularly obtained with other lithography techniques. The bound on resolution limits the range of potential applications and is a fundamental limitation for more complex designs. Several methods have been proposed to increase the resolution limit, however in general these methods significantly complicate the fabrication process [17, 21]. Geometrical artifacts in TPP can arise from misplacement of the polymerization voxel [17, 24], translation of the design into a voxel-by-voxel printing protocol which can be accomplished by the printer [25, 26], step-wise layered 3D printing [27], and changes to the structure during the post-printing development. The latter include shrinking [28, 29], striation [30], and general deformation. Some examples of these types of fabrication non-idealities or artifacts arising from the TPP deposition and development process are shown in Figure 1. Fabricated artifacts can be local or global in scale. Local artifacts due to the rounded voxel shape and striation between parallel layers, respectively, are shown in Figure 1B and C. Figure 1D shows an artifact where regular arrays composed of multiple nanopillars exhibit a global trend affecting the color resonance [16]. This is an example of a global artifact since it involves many individual nanostructures. Additionally, since TPP is an additive manufacturing process directly depositing material into the final configuration, fabrication artifacts not common in subtractive lithography methods can be introduced including spatiotemporal variation of chemical or material properties such as polymerization density, and the inclusion of oligomers (”blobs”) or voids in the final structure [31, 32].

Fabrication process improvements are one solution to mitigate fabrication limitations and artifacts, and as such are the main focus of many current TPP investigations. Recent advances in TPP technology have centered on optimizing the photo-polymerization chemistry and processing technique to improve throughput, cost, and minimum feature size [21]. However, such studies can often be time-consuming and expensive, and the search for new photo-polymerization chemistries is often challenging.

## 3 Physics-informed design approaches

Alongside refinement of the deposition process, we propose that many of the challenges outlined above could be mitigated by modifying simulation and design methods to better encapsulate the physical realities of device fabrication in the design process. In different cases, the functional dependence of fabrication artifacts upon the process parameters can range from simple to complex. For some simpler fabrication-based artifacts with clear functional dependence on system parameters, one way to compensate for induced artifacts is via offset algorithms (OA) [22, 33]. In the OA process system parameters are varied and their effects on the resulting structure and optical resonances are measured. The relationship between input parameter and response is then used to calculate systematic offsets to the input to minimize known artifacts. For example, Lim et al. utilized a OA to correct for deposited structure size discrepancies in a TPP nano-replication process [33]. A systematic offset was calculated based on both the designed size and the voxel size. However, in some cases the dependence of fabrication artifacts upon the process parameters can become complicated, making the determination of offsets difficult. Utilizing the idea of systematic offsets from OA algorithms, in these cases we propose that modern inverse design methods can be used to implement more complex compensation schemes which would be difficult to capture with traditional methods. In particular, employing physics-informed inverse design methods that exploit information from underlying theoretical and observed physical relationships could be a powerful tool to correct for a range of complicated fabrication artifacts.

In recent years inverse design—the computational design of nanophotonic systems to engineer a particular electromagnetic response—has helped to reshape the landscape of nanophotonic design, leading to a revolution in the range of potential device capabilities [34, 35]. The cornerstone of these approaches is computational optimization schemes, including gradient-based approaches and more sophisticated algorithms such as genetic algorithms [36, 37], adjoint methods [38], and machine learning [39, 40]. In these algorithms, the sensitivity of the electromagnetic response to changes in the design parameters is calculated from multiple nanophotonic simulations [41]. In the case of machine learning this typically involves the generation of a large simulated data set. The design parameters are updated in response to the loss function, a function that calculates a metric based on the difference between the proposed and targeted electromagnetic response. The loss function typically employs a nanophotonic simulation and can contain terms corresponding to equations and relationships based on the specific problem at hand.

Nanophotonic optimization is generally difficult due to large parameter spaces, correlations in the response between multiple distinct parameters, and highly nonlinear relationships between structure and optical response [34]. To render the problem more tractable, current design methodologies typically ignore many details of fabrication processes in favor of idealized models and structures such as smooth, homogeneous elements. In reality, the fabricated structure may be far more complex. Although designs created in this process are physically and computationally simpler, they do not necessarily relate to directly fabricable structures. Artifacts introduced by subsequent fabrication can lead to sub-optimal electromagnetic response, especially in the case of high quality-factor resonances [42, 43]. While geometric inconsistencies can sometimes be accommodated by increasing model complexity, this can drastically increase the computational cost. Furthermore, more detailed fabrication-based effects often involve a range of physical interactions on multiple scales which can be difficult to incorporate in a single simulation [44].

We propose that physics-informed optimization and design methods are especially well-suited for tackling the problems outlined above. Physics-informed methods build upon traditional methods by incorporating prior knowledge about the underlying physics and observed data to strategically improve the function of an algorithm [18, 45, 46]. Although the principles of physics-informed methods can be used to enhance a wide range of optimization and design algorithms, the vast majority of implementations have been for machine learning algorithms. Note that all of the techniques described below can be utilized in a machine learning context. Additionally, some methods specific to machine learning will be discussed in Section 3.1.

Physics-informed methods have been implemented in a diverse array of scientific and engineering fields, including metamaterials and photonics [47, 48]. Several works have recently demonstrated the potential advantages of physics-informed methods over traditional methods, including faster convergence and `data efficiency (which is especially important in many scientific problems), as well as increased interpretability and generalizability in machine learning models [49–57].

In traditional optimization, the internalization of *a priori* physical information relevant to the design process, such as approximations, conservation laws, and parameter relationships, is either generalized from the data set or ignored. With physics-informed methods, the optimization algorithm can utilize provided physical knowledge to effectively decrease the complexity of the optimization problem while creating solutions that better correspond with physical reality. Similar to how we as humans understand complex physical systems, a hybrid approach utilizing analytical, computational, and observational elements is often useful to guide our interpretation of otherwise incomprehensible data sets.

**Figure 2:**
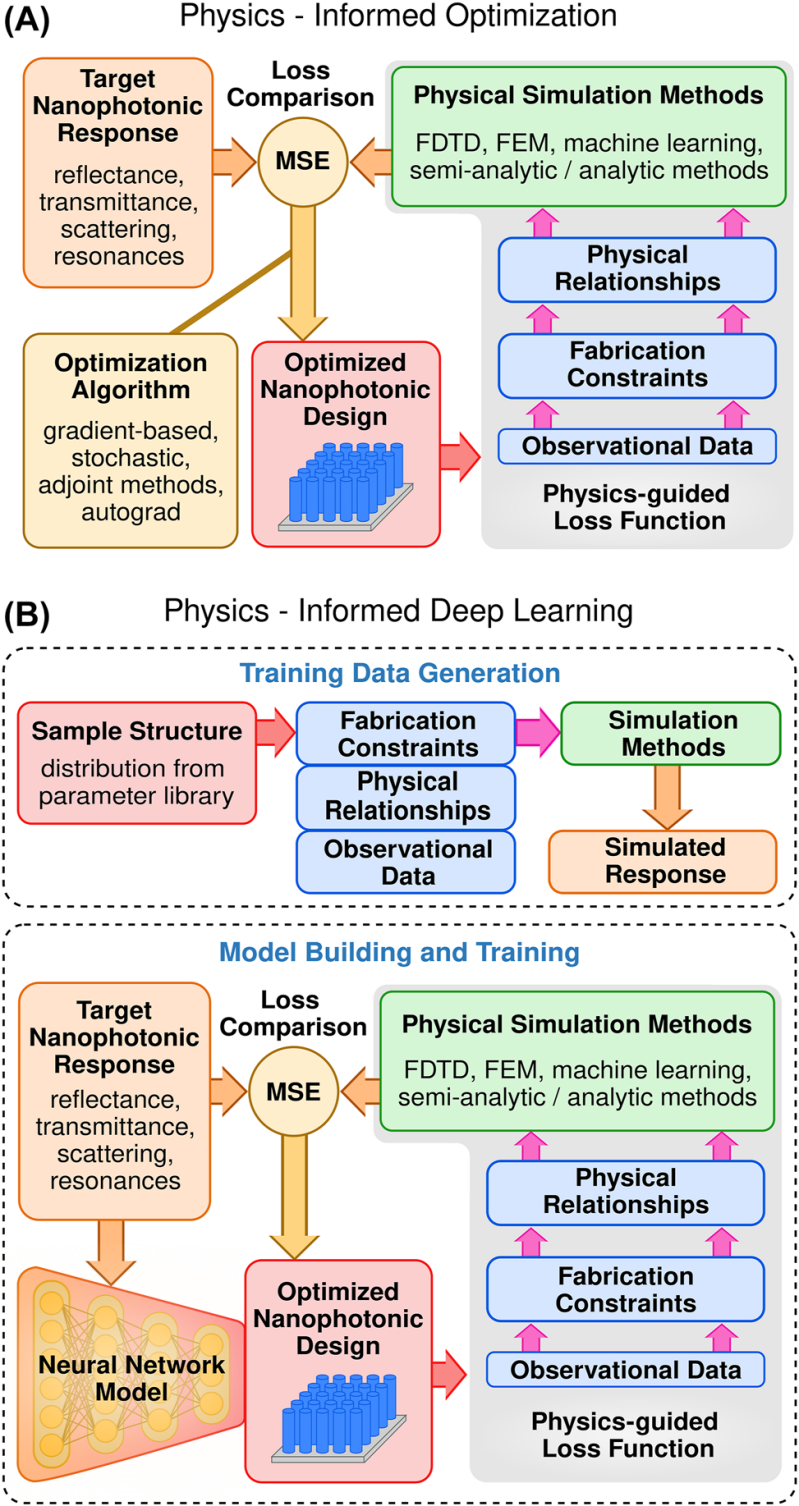
Schematic detailing the physics-informed approaches to nanophotonic design optimization. (A) Traditional optimization algorithms (yellow) can be used to tune nanophotonic designs. In physics-informed methods, physically relevant information (blue) can be incorporated into the loss function. (B) The physics-informed nanophotonic deep learning directly incorporates physically relevant information in the training of machine learning models. This can be implemented in the training data generation, or in the network training loop. Note the procedural similarities between the deep learning and general optimization approaches.

Nanophotonic design optimization for additive manufacturing has many attributes amenable to physics-informed optimization. Importantly, a well-developed theoretical framework for photonics exists, together with a wealth of experimental data [58]. In many cases, this extends to the physico-chemical interactions associated with the polymerization process and the internal structure of the polymerized material [59]. Since multiple structures can typically be fabricated and analyzed quickly, machine- and process-specific observational data can be readily obtained. Finally, fabrication errors are usually reproducible, even if they can be difficult to correlate with a specific parameter. Physics-informed techniques have been previously implemented in a wide range of other fields, from manufacturing to climate modeling, so many techniques currently exist which could be applied to photonics [45, 56, 60], [61], [62], [63], [64], [65], [66]. There are several avenues to incorporate physically relevant information into a design algorithm, which are explored in detail in the next sections: physical relationships among parameters, transformations based on observational data, and physically-determined parameter constraints. These techniques can be used individually or in combination to constitute physics-informed optimization methods, as illustrated in Figure 2A.

### 3.1 Physical relationships

Physical relationships relating to nanophotonic systems may include, for example, the laws of electromagnetism, analytic descriptions of particular resonances, or other known relations [67–69]. In the specific context of additive manufacturing we can also consider the chemical aspects of the photo-polymerization process, secondary reactions such as quenching mechanisms and reactant depletion, and relations involving the polymerized material [70–72].

In general, these kinds of relations can be useful in a variety of ways: as a means to augment the loss function by calculating new aspects of generated structures, for including information into the optimization process which goes beyond the scope of simulation method, and for introducing information which is difficult to generalize from data sets. Other physical relationships can be used in tandem with different methods, for example in calculating parameter constraints as described below. Physical relationships can present in a variety of forms including PDEs/ODEs, inductive biases, general equations, and symmetry operations [18, 73], [74], [75]. The specific implementation will depend on both the type of problem and the representation of the physical law. However, most physical relationships can be inserted into the loss function as transformations of predicted physical parameters, or implicitly imposed by controlling the functional form of the output [76]. Examples of the latter include setting up the optimization problem to predict parameters of a differential equation, or predicting parameters of functions which match the known symmetrical form of the physical problem [77–79].

Several recent works have demonstrated the effectiveness of this approach. For example, in a recent work Chen et al. created a machine learning based surrogate electromagnetic solver with a custom loss function to ensure compliance of the calculated fields with Maxwell’s equations and used Ampere’s law to calculate electric from magnetic fields [50, 69]. This enabled a significant speedup in the optimization time by increasing the physicality of the proposed solutions. Liang et al. created a physics-guided neural network directly utilizing coupled mode theory to improve the inverse design of photonic–plasmonic waveguides [80, 81]. This allowed them to design complicated photonic structures while utilizing compact neural networks, obtaining high accuracy and simplifying and accelerating the design problem. In another example Khatib et al. designed a “deep Lorentzian neural network” machine learning approach to predict effective medium parameters for a dielectric disk metasurface [82]. Causality, a property typically neglected in effective medium approximations, was enforced in the model prediction by restructuring the network output to predict the effective permittivity and permeability as a series of Lorentzian oscillators. These functions are known to satisfy causality, ensuring compliance in the network output.

In an example from manufacturing, Liu and Guo created a hybrid machine learning and physical modeling system to calculate cutting energy in steel milling by combining physical models of the cutting mechanics with machine learning methods [83, 84]. The physics-informed model showed a significantly reduced error compared to the base model. In nanophotonics, Chen et al. have detailed the benefits of physics-informed machine learning methods in computational inverse design, including photonic metamaterial design and Mie scattering [67]. We expect that physics-informed methods will be especially useful in the context of difficult metasurface design problems, such as in the design of Huygens metasurfaces in which the total response can depend upon long-range interactions between nanophotonic elements [85].

### 3.2 Observational data

Often a fabricated structure will have a range of aspects which are not immediately identifiable with a known physical relationship. This may include, for instance, observed fabrication artifacts associated with either geometrical or internal (polymer, chemistry based) features, and experimentally verified parameter trends over time and space [17, 86, 87]. When attempting to optimize for fabrication-based artifacts, additions based on observational data are often important. In TPP fabricated devices, the observed data can be either local to a single structure or global to a larger nanophotonic array. Observational data additions are generally useful when attempting to tune simulation models or fabricated outputs to a specific machine or deposition type, responding to reproducible artifacts. Artifacts can generally be identified by fabricating and experimentally measuring a number of structures. The observed trends can then be either interpreted in analytic form or incorporated by the inductive capacity of an optimization or machine learning method. Fewer structures can be fabricated for artifacts where the observed trends can be easily explained with few process variables, however more complex or unpredictable trends can require experimentally verifying a large range of structures which may become time and cost intensive [18].

Depending on the specific problem, artifacts may be best described by a discrete relationship or a distribution of fabrication results [88]. Discrete relationships (such as regularized post-processing shrinkage) can be incorporated into the loss function as transformations on the design parameters. For stochastic artifacts (such as randomly distributed voxel misplacement errors), optimization methods capable of handling stochastic problems are required [89–91]. Global observed trends can be included by performing the simultaneous optimization of an entire nanophotonic array, or by considering offsets to the design of a single structure based on its spatiotemporal placement during fabrication. Some artifacts can be easily generalized and incorporated if they have a calculable effect on a systematic parameter. However, more complex relationships may rely on techniques such as machine learning to generalize the observed trends [92].

As an example, Du et al. recently utilized experimental data on defect formation in metal additive manufacturing to build a mechanistic model based on process variables [65]. This model was then incorporated into a physics-informed framework to predict and reduce common structural defects. In another example, Wenzel et al. utilized physics-informed machine learning to increase the reliability of fused-filament fabrication, a difficult problem due to stochastic dependencies on the process parameters [93]. Both theoretical and experimental data were used to optimize neural network models to mitigate typical fabrication errors.

### 3.3 Fabrication constraints

Knowledge of the physical aspects of the fabrication system can be used to provide constraints on the acceptable ranges of design parameters. Fabrication constraints are different from observational data and physical constraints since they deal with fundamental limitations due to the fabrication process rather than introduced artifacts or theoretical limitations [94]. However, the underlying knowledge in the constraint can be derived via theoretical or empirical means, similar to those introduced above. Constraints may include, for instance, minimum feature size or maximum aspect ratio structural limits, constraints based on known effective ranges for specific resonance models, or other structural limits based on chemical aspects of the polymer [95].

Constraints are typically implemented as a loss function penalty for out-of-bounds predictions, allowing the implementation of “soft” constraints based on the severity of the penalty [96]. Constraints on parameter ranges can also be directly incorporated by utilizing analytic equations such as boundary conditions, or indirectly built into the loss function by passing predicted variables to constrained model structures, such as range-bounded functions. It should be noted that often the nonlinearity of the function can often have an effect upon the optimization.

While constraints specific to fabrication are not as widely explored in the literature, the incorporation of physically motivated constraints has been shown to be effective in a range of optimization problems. As an example, Chen et al. recently proposed a machine learning model to characterize deep carbonate reservoirs from seismic data [97]. This model utilized four types of physically motivated constraints: continuity between data points, boundary constraints, constraints based on the spatial position of reservoirs, and category constraints to balance the data set. The performance of the model was significantly improved by utilizing physics constraints. Lu et al. have investigated several methods for employing hard and soft constraints for solving holography and fluid flow inverse design problems. PDE boundary conditions are implemented as the system constraints in a physics-informed machine learning context. Including constraints was found to produce smoother designs for non-unique optimization problems [57].

## 4 Machine learning specific approaches

The physics-informed principles discussed above can, in principle, apply to a wide range of optimization techniques within nanophotonics. However, the dominant application of physics-informed techniques is in the context of improving or augmenting machine learning approaches. Several recent works utilizing physics-informed machine learning (PIML) approaches are compared in Table 1. We have focused on works related to nanoscale photonics and additive manufacturing. For more advanced and comprehensive discussion on PIML approaches we direct the reader to several recent reviews on the topic [18, 45, 56, 98], [99], [100].

**Table 1:** Recent implementations of physics-informed machine learning.

Publication	Category	Physics-informed strategies	Results/advantages
Chen et al. [50]	Physical relationships (laws)	Custom loss function based on Maxwell’s equations and Ampere’s law	Increased optimization speed and physicality in predictions
Liang et al. [80]	Physical relationships (resonances)	Neural network directly incorporating coupled mode theory	Increased accuracy and optimization speed
Khatib et al. [82]	Physical relationships (parameter relations)	Neural network output constraint in terms of physically relevant functions	Increased performance and physically meaningful output
Liu and Guo [83]	Physical relationships (modeling)	Physics-informed loss function including models of steel milling	Significantly reduced prediction error
Du et al. [65]	Observational data	Mechanistic modeling of process parameters and incorporation into a machine learning model	Better prediction of balling defects and understanding of mechanisms
Wenzel et al. [93]	Observational data	Incorporation of process parameters and physics informed pre-training of a neural network	Faster prediction time and increased reliability
Chen et al. [97]	Fabrication (physical) constraints	Included several types of physically motivated constraints on a neural network model	Significant performance improvements
Li et al. [57]	Fabrication (physical) constraints	Employed hard and soft constraints on holography and fluid flow PDE problems	Produced smoother designs for non-unique problems

Machine learning is an increasingly important tool in nanophotonic design, and many recent works have demonstrated machine learning as a powerful means to solve the inverse design problem in nanophotonics [40, 48, 101, 102]. Machine learning utilizes data-driven techniques to automate the internalization of complex information for prediction and decision-making tasks [103, 104]. It is especially well-suited for dealing with problems where the underlying relationships are complex or hard to generalize with traditional models. Within machine learning, deep learning approaches have been widely explored in the context of nanophotonics [40, 48]. In deep learning multilayer neural network models are constructed and trained to produce an internalized representation of the underlying patterns and relationships in a data set [105, 106]. As with traditional optimization, deep learning relies on a loss function to quantify the performance of the model.

Although machine learning methods excel at demonstrating statistical correlations, they are often notorious for ignoring the physicality of the optimization problem. This can lead to spurious predictions and overfitting, and engender low generalizability of the model [107, 108]. Physics-informed methods are one way to counter this issue, allowing the model to access the underlying physics of the learning problem. PIML extends upon traditional machine learning by integrating physical knowledge directly into the construction model and training procedures. A schematic representation of integrating physical information into the loss function of machine learning models as described in the previous section is shown in Figure 2B. PIML has several benefits over traditional machine learning. By utilizing the extended physical information, models can be built using less training data, and trained more efficiently [109]. PIML models can also possess greater generalizability and portability to new parameter domains, as well as greater interpretability and improved robustness when faced with noisy or uncertain data [73, 99]. However, PIML requires knowledge of the physical underpinnings of an optimization problem, which is not always accessible. Additionally, imposing physical constraints can reduce the innate generalization ability of deep machine learning in cases where the physics is not fully captured.

Given the optimization capabilities of machine learning and the relative ease at which many of the above techniques can be implemented in a deep learning framework, machine learning is situated as a premier tool for physics-informed optimization of nanophotonic systems [18, 49, 100]. However, the physics-informed methods described above can typically be utilized in both machine learning and traditional optimization schemes. The decision to utilize machine learning or traditional optimization should be based on the specific problem at hand. Machine learning excels at solving problems involving complex relationships which are difficult to generalize. For complex problems with many parameters, or for many evaluation instances, machine learning can typically produce results faster than comparable optimization methods [110–112]. However, large training data sets are typically required which can be computationally or experimentally intensive, and some neural networks require long and expensive training times to achieve their stated performance [113, 114]. For optimization problems involving fewer parameters and evaluation instances, traditional optimization may be more efficient than a deep learning approach. Additionally, in PIML the computational efficiency of the included models or simulation methods can have a notable impact on convergence time [115]. This should be taken into account when deciding to utilize PIML methods, and alternative simulation types or other physics-informed components supporting computationally efficient gradients such as automatic differentiation should be preferred [116, 117].

In addition to the methods outlined previously, there are several specific physics-informed machine learning techniques which are primarily utilized in a deep learning context. These can be broadly classified into three categories: physics-informed manipulations of the model input, components and architecture [56].

Model input manipulations involve pre-processing data at the input of a neural network model to extract or augment with physically meaningful information. This can involve any of the main categories mentioned above including static transformations such as Fourier transforms and symmetry operations, physically relevant simulations, and augmentation with physically relevant parameters [118, 119]. Input manipulations can have the effect of guiding the training by increasing the breadth of input data to the model, or highlighting specific relevant features which would otherwise be difficult to discern.

Physics-informed manipulation of the model components and architecture involves designing the internal machine learning model structure to encapsulate or tune the model to better respond to physically relevant aspects of the optimization problem. Machine learning models support a number of components on which this technique can be used, including: activation functions [120, 121], individual layer and model types [122–124], parameter initialization [125], and deep learning layer and model structure [126–129]. In each case the machine learning model components are chosen to reflect a specific aspect of the physical system. For example, Howland and Dabiri [120] recently created a machine learning model using an activation function mirroring the nonlinearity observed in a relevant physical parameter. This allowed the model to better encapsulate the physical problem, reducing prediction error. In the context of layer and model structure, Gao et al. [122] recently designed a method to utilize convolutional neural networks with PDEs on non-uniform grids which are common in a range of physical problems.

## 5 Conclusions

Additive manufacturing is a promising technology for widespread applications in visible photonics. However, it faces several challenges relating to the design of realistically fabricable structures. We propose that physics-informed methods—embedding physically relevant information within optimization or machine learning models—are particularly well positioned to create design and optimization frameworks capable of rising to these challenges for visible photonics.
